# Implications of Chronic Opioid Therapy on Perioperative Complications and Long-Term Surgical Recovery

**Published:** 2019-08-29

**Authors:** Da Liu, Matthew DiMeglio, Michael DiMartino, Jihane Hajj, Maria Mukhanova, Karima Rai, Mazell Winikor, Krzysztof Laudanski

**Affiliations:** 1 Department of Anesthesiology and Critical Care, Hospital of the University of Pennsylvania, Philadelphia, PA, USA; 2 DO/MBA Student, Philadelphia College of Osteopathic Medicine, Philadelphia, PA, USA; 3 Department of Biology, University of Pennsylvania, Philadelphia, PA, USA; 4 Department of Cardiology, Penn Presbyterian Medical Center, Philadelphia, PA, USA; 5 Department of Nursing, Widener University, Chester, PA, USA; 6 Department of Biology, Drexel University, Philadelphia, PA, USA; 7 Leonard Davis Institute for Health Economics, University of Pennsylvania, Philadelphia, PA, USA

**Keywords:** Opioids, mu receptor, kappa receptor, mu-delta receptor, morphine, hydromorphone, fentanyl

## Abstract

With chronic opioid use becoming an increasingly common occurrenceamong the general population, perioperative specialties must adapt to the physiologic changes caused by long-term opioids. However, data on the clinicalanesthetics implications of long-term opioid use is scarce. This review intends to survey the literature addressing the molecular mechanisms of long-term opioid use as well as their interaction with various organ systems.

## Background

Opioid use and abuse has been consistently increasing in the United States over the past decade with an estimated 4.3 million adults regularly taking opioids [1,2]. Of these individuals, it is estimated that up to 29% exhibit opioid misuse and 12% exhibit opioid addiction behaviors [2]. Concomitant abuse of heroin and the synthetic opioid, fentanyl, have contributed to a recent dramatic increase in overdose deaths. Increase in illicit substance abuse in general, increased incidence of chronic pain, pressure on providers to ensure patient’s optimal experience in hospital, the introduction of pain as fifth vital sign and, most importantly, the influence of pharmaceutical companies has led to a “perfect storm” in the USA [4]. Opioid use has also become a global epidemic, with areas such as Eastern Europe, North Africa, and the Middle East exhibiting high prevalence of opioid-dependence [5]. The use of opioids have also shifted from an acute therapy to chronic management of pain, and the detrimental effects of long-term opioid therapy are beginning to be described in the literature [2,6,7]. Overall, the exposure of the public to opioids is increasing and the nature of opioid ingestion is changing towards chronic intake. Furthermore, some of the exposure takes place at earlier ages and even during fetal development. The effect of chronic opioids exposure during at the developmental age is profound yet the scientific community has yet to grasp its clinical impact.

It is a matter of curiosity to ask how the health consequences of chronic intake of opioids will affect medical decision-making during anesthesia and surgery. The focus of this review is to examine the biological effects of prolonged exposure to opioids as it relates to their significance in adapting an anesthetic plan. We will focus on exogenous opioids and not comment about the natural ligands of opioid receptors. We will not address the issue of prolonged use of ketamine and cocaine and other pharmacological compounds with mixed receptor mechanisms, except methadone. The latter compound has several quite unique properties, but its importance in management of chronic pain warrants inclusion. Opioids will be defined as the compounds interacting solely with mu (μ), kappa (k), and delta (δ) receptor. We will not investigate the chronic effects of sigma (σ) receptor stimulation since it is not triggered by endogenous opioids. Also, we will not address the biological importance of nociceptors or the opioid growth factor receptor (OGFR or ζ receptor) considering ongoing controversies related to those receptors’ pharmacokinetics and highly dynamic evolution of their science. Finally, we will focus on long-term consequences of opioid use defined as longer than three months of consistent use. This definition is somewhat arbitrary, yet most of the patients will recover from primary illness in three months. Patients who are on opioids at that time are more likely entering the chronic phase of the drug use. The intention of this review is to demonstrate several observations potentially affecting the administration of anesthesia during surgical procedures.

Our review is intended for perioperative medical specialists. Often, for the anesthesiologist, the issue of chronic opioids use becomes evident during the perioperative management of pain. Considering significant body of evidence we will not discuss how chronic opioids use will affects perioperative pain management unless it is related to molecular level mechanisms. However, opioids have several properties outside pain modulation. Most of these effects are often discounted during perioperative delivery of the anesthesia. The increasing emphasis on rapid recovery, durability of the treatment, and long-term outcome of medical interventions may soon put anesthesiologist in the situation to consider the long-term effect of opioid use on perioperative planning.

### Methodology

The purpose of this review is to critically review data on the effect of chronic opioids use on the performance of several systems. A PubMed, International Scientific Indexing (ISI), and Web of Science search was conducted utilized search terms and synonyms of long-term, opioids, and immunologic effects. Non-English language articles were excluded. Bibliographies of retrieved studies were searched for other relevant studies. Articles were reviewed for their contribution to understanding the long-term effects of chronic opioids, with priority given to clinical trials, large retrospective studies, and prospective studies in the basic sciences. A total of 56 original research studies were reviewed for their relevance and utilized in this review. The following themes are addressed in this review: 1) The effect of chronic opioid use on opioid receptors 2) The effect of chronic opioid use on the regulation and performance of organ systems.

### Receptor Mechanisms and Prolonged Stimulation

Opioids act through seven-transmembrane G-coupled receptors. Binding of the ligand results in the release of guanosine diphosphate (GDP) from the G α-sub-unit followed by binding of guanosine triphosphate (GTP). This replacement results in significantly lower affinity of to the Gßy and separation. Once lipid anchors are cleaved, the active G subunit inhibits adenyl cyclase activity resulting in an increase in cyclic adenosine monophosphate (cAMP). In addition, hyperpolarization of the cellular membrane is mediated by activation of inward rectifying potassium channel (K_ir_). cAMP acts as a secondary messenger activating protein kinase A (PKA). In turn, PKA phosphorylates cAMP binding responsive element (CREB). CREB is critical in regulating several genes which are critical in long-term potentiation and synapse formation. This is a “classical” series of molecular event after opioid binding [8]. However, prolonged exposure of opioid receptor triggers different molecular events from short term stimulation.

During chronic opioid use, the opioid receptors are exposed to repeated activation and/or prolonged stimulation. Typically, this will trigger several mechanisms altering the physiologic effect of opioids, in addition to changes in pharmacokinetic performance. One of the mechanisms regulating exposure of opioid receptors to ligands is phosphorylation. One study found that mice chronically exposed to morphine exhibited decreased PKA-induced phosphorylation of μ-opioid receptors leading to a 50% downregulation in μ-opioid receptors, revealing a potential mechanism for morphine tolerance [9]. The second mechanism regulating chronic exposure to opioids is ß-arrestin-mediated endocytosis of the receptor reducing the effective sensing of opioid ligands [10,11]. It is important to note that the total number of receptors remains the same in the brain since trans-location, not digestion, of receptors is primary mechanism [12]. Downregulation is also not a simple process of reducing the number of open to stimulation receptors, but rather qualitatively changes the physiological response to opioids via a variety of mechanisms. In addition to phosphorylation, the chronic exposure to opioids leads to preferential formation μ-δ heterodimers in critical areas of the central nervous system [13,14]. Formation of heterodimers significantly impairs ligand binding and reduces some of the downstream effects of the heterodimerized receptor. Milan-Lobo *et al.* (2013) demonstrated the significance of heterodimer forming on pain perception and the role of the antagonists of μ-δ heterodimers on improving the reduced analgesic effects of chronic opioid use [15].

The translational importance of these findings warrants further research. Theoretically, these mechanisms (phosphorylation, endocytosis, heterodimer formation) may prevent hyperactivation of cAMP due to chronic receptor stimulation. cAMP hyperactivation would trigger several downstream effects like hyperactivation of extracellular signal-related kinase (Erk), a predominantly inflammatory pathway. However, the activation of Erk is significantly downregulated during chronic exposure of opioids, suggesting an interplay of other mechanisms. Understanding why Erk is downregulated in this particular situation is critical to understand the immunoinhibitory effect of opioids, considering that Erk is an essential component of inflammatory pathways [16]. A similar mystery is encountered in case of differential regulation of *TSP1 versus TSP2* release by chronic vs. acute morphine exposure [17].

### Role of Chronic Opioids in Regulation of Various Systems

#### Nervous system

Chronic exposure to opioids induces loss of inhibition of neurotransmitter release in the brain. However, this effect decreases over time [18]. Functional significance and clinical relevance of this finding has not been defined. A recent study demonstrated that modulation of GABA pathway by cannabinoids is affected by chronic uptake of opioids. This effect is mainly present in the grey paraductal area [19]. This area is critical for several homeostatic functions but the clinical correlation is missing from the study.

It is unclear if the seizure threshold is altered in a patient taking chronic opioids. A case report was published that demonstrated controlled-release oxycodone as a potential cause of seizure in an individual with a pre-existing seizure disorder [20]. However, a more extensive subsequent study did not comment on the decreased seizure threshold in patients using prolonged narcotics to control chronic pain [21]. Animal studies showed a lower threshold for seizure in morphine-dependent animals [22].

#### Immune system

The effect of chronic opioids on immunity is relatively well investigated. Opioids tend to be immunosuppressive via direct and indirect mechanisms in the case of exogenous administration. In contrast, endogenous opioids are immunomodulatory [23,24]. This was a significant bias in analyzing specific aspects of opioid-induced neuro-immunomodulation in the past [25]. The indirect mechanisms of immunosuppression rely on a complex interaction with the endocrine and nervous system further clouding the effect of chronic opioids on immunity. However, these effects will not be covered in this review.

There is substantial evidence that opioids directly affect several leukocyte populations [26]. However, the relative significance of direct *versus* indirect mechanisms continues to be debated. *In vivo,* the immunological effects of opioids are mostly maintained via a central mechanism since μ-receptor knockout mice did not show any immunological effect of intrathecally administered morphine [27]. In contrast, studies incubating leukocytes with opioids *in vitro* are often challenging to translate into clinical practice. In one example, the gene expression of leukocytes is severely affected by exposure by morphine though authors use supraphysiological concentrations, and the duration of exposure was short [28]. However, it remains unclear how these conditions relate to the duration of exposure and drug concentrations seen in the common clinical scenario such as an individual on chronic extended-release opioid therapy. The specific, and most frequently cited, peripheral effects of opioids include suppression of B and T cells proliferation, reduced activity of NK cells, diminished immunoglobulin production, responsiveness to lipopolysaccharide, generation of competent dendritic cells, and switch of CD4+/CD8+ T-cell proportion [6,25,29,30]. Some of these effects emerge after only 8 days of morphine exposure [30]. It is unclear how these effects are maintained, evolved, or dissipated during chronic opioids exposure. Efforts on linking the opioid-mediated immunological alterations to clinical outcomes are difficult considering that chronic opioid intake is often complicated by other co-morbidities. Nevertheless, in a landmark study, it was demonstrated that opioids affect the expression of chemokines, critical molecules involved in trafficking of the immune system cells, and HIV virus uptake [31]. Other showed persistent mycobacterial infection in chronic opioids [32,33]. In the latter study, however HIV-infection was a confounder [33].

Interestingly, the immunomodulatory effect of opioids depend on compound type suggesting additional mechanisms at play [34]. Hydromorphone and buprenorphine are practically devoid of immunosuppressive properties while morphine, sufentanil, and fentanyl are quite effective immunosuppressants. Noteworthy, their immunological properties do not align well with analgesic potency [34]. This raises the question of opioid selection based on immunologic potency in clinical situations such as cancer surgery. The long-term immunomodulatory effect may benefit a patient, while lower analgesic potency can be compensated with dose titration or adjunctive treatment. However, the evidence for clinical significance of immunological properties of different narcotics in perioperative period is rather weak. Also, most of the studies demonstrating different immunological potency of opioids were done *in vitro* or animal models. These studies have inherited limitations due to the difference in human and animal physiology especially as it pertains to opioid molecular mechanisms. Few human studies investigated the immunologic significance of different exogenous opioid ligands but failed to address the question of clinical relevance. Only one randomized clinical trial compared two different opioids with respect to immune system performance [35]. A study by Neri *et al.* (2005) showed no effect between methadone and buprenorphine on serum level of IL-1ß, TNFα, and frequency CD14+ cells [35]. However, the study utilized crude indices of immune system performance, was underpowered, and provided no clinical correlations.

#### Endocrine system

Prolonged exposure to opioids affects glucose metabolism on several levels. Some of these effects include decreased production of insulin-like hormone type 2, growth hormone, and cortisol [36]. This contrasts with acute exposure to opioids, which stimulates the release of growth hormone and insulin growth factor 1 [37]. This is most likely secondary to release of preformed hormones. In contrast, chronic exposure leads to reduced expression of mRNA, leading to depressed serum levels. This depression of hormonal RNA was not universal, with some subjects having much more profound effect than others [36,38]. Considering that interindividual variability in opioids metabolism has significant clinical relevance, it is an interesting question if the difference in opioids metabolism has a real effect on glucose metabolism.

The effect of chronic opioid intake to the urinary free cortisol excretion demonstrated a significant depression in cortisol synthesize. However, such an effect takes between one to twelve weeks of opioid intake to develop. Significant confounder here is the effect of co-existing chronic stress on the serum level of cortisol and ACTH. However, it is sound to conclude that chronic opioids may blunt the stress cortisol response with some theoretical significance during the delivery of anesthesia. This observation was reinforced by several cases report [39]. Currently, opioid induced adrenal deficiency is a recognized clinical phenomenon. Approximately, 20% of chronic opioids user will experience its symptoms but most of them are related to fatigue and poor emotional status [40,41]. These observations suggest though that there is a risk of catastrophic adrenal crisis during anesthesia in chronic opioid intake.

Thyroid axis was mostly undisturbed, or poorly studied, in chronic opioids use despite their importance to overall homeostasis [36]. Production of procalcitonin was unaltered as well as demonstrated in a single study [42].

One of the best-established effects of chronic opioids is its effect on sex hormones [43]. Opioids related sex hormones endocrinopathy emerges in more than 50% of women treated with chronic opioids leading to amenorrhea [36,44,45]. Hypogonadism concomitant to a depressed level of testosterone was also frequently reported [43,44,46]. However, sex hormones interplay has rarely an influence on anesthetic plan.

Regarding sex-dependent differences in analgesia, hyperalgesia, tolerance, and dependence to opioids, the female sex has been woefully underrepresented in studies [47,48]. However, there is a growing body of literature identifying such differences [48]. Differential spinal analgesia was demonstrated in rodents where females required both μ and k receptor stimulation for analgia, while males required only μ receptor stimulation [49]. The same pattern was observed in supraspinal analgesia where male rodents experienced greater analgesia from viscerosomatic pain than their female counterparts during systemic and intraventricular, but not intrathecal, administration of opioid [50].

Summarizing, chronic opioids have at least the theoretical potential to affect the outcome of the anesthesia and surgery consider their potential to blunt stress response, affect glucose metabolism, and cause sex-dependent pain responses.

#### Cardiovascular system

Data regarding the direct effect of opioids on the myocardium is limited [51]. Consequently, the clinical relevance of acute opioid administration on cardiovascular performance is likely minimal and mostly mediated via collateral vagal reflexes of histamine release [52]. Furthermore, studies investigating the direct effect of chronic opioid intake are virtually non-existent. The consensus is that opioid receptors in the heart are not involved in mechanical heart performance, but serve an important role in conditioning [47,49,50]. However, most of the studies were conducted during acute exposure of exogenous opioids to δ and k opioid receptor [55,56]. The formation of heterodimers is one of the most important mechanism of how opioid receptors play in cardipotection. This effect is dampened by ß_2_-ad-renergic receptor stimulation [56].

However, findings from bench research do not necessarily translate into clinical observations. In retrospective study, Carman *et al.* (2011) demonstrated an increased risk of myocardial infarction in a patient taking chronic opioids as compared to COX-2 [57]. The excess risk was present despite adjustment for several confounders. The large study sample also strengthens this retrospective observation. However, the authors were unable to conclusively attribute the increased risk of acute coronary syndrome in chronic pain users [57]. More recently, a large retrospective study found that chronic opioid therapy was associated with an increased risk of cardiovascular deaths, confirming prior studies [6]. This was the second reason for increased mortality among chronic narcotics users. Again, the inherent limitations of a retrospective cohort study precluded the ability to address the underlying cause of increased mortality in chronic opioid users.

However, what is interesting about these findings are that they are contradictory to laboratory studies. *In vitro* investigations and animals studies demonstrated that opioids are able to condition myocardium to ischemic events quite efficiently [58,59]. Therefore, the excess mortality secondary to the cardiac event cannot be easily explained. It is possible that the reduced ability to respond to stress via hormonal surge, increased blood glucose level, and worsening in lipid profile may accelerate the progression of cardiovascular disease and increase the risk of death from cardiovascular disease. Future research is needed to address rationales behind the contribution of chronic opioid use to cardiovascular events and death.

The most common effect of opioids is related to their properties of prolonged QTc in the case when the patient is on methadone. Despite the prolongation of QTc, the mortality effect of methadone induces QTc prolongation are limited [60,61]. Increased awareness of this finding is important since a patient may receive another QTc prolongation drugs during anesthesia delivery (metoclopramide, ß-blocker, ondansetron).

#### Respiratory system

Anxiety related to respiratory depression or his-tamine-mediated bronchospasm has long dominated physician perception of the influence of opioids on the respiratory system [62]. With the rise of opioid-induced death, research into opioid-mediated respiratory depression [63]. It seems that virtually no studies assessed the effects of prolonged exposure to opioids on the respiratory drive in respect to clinical outcomes. However, the potential development of tolerance is met by increased sensitivity due to the age, increased doses of opioids and declining health status as seen in case of COPD patients [64]. The area is further complicated by an ability of small dose morphine to relieve dyspnea [65]. This beneficial advantage is accompanied by increased mortality suggesting that the margin of error in dosing opioids in the patient with compromise respiratory status is too narrow to finesse clinical benefit [66]. Also, the majority of the therapies are focusing on the use of compounds to stimulate breathing to overcome depressive effects of narcotics or adaptive servo-ventilation (ASV) [67]. In this respect, studying long-term modulation of respiratory drive by opioids does not appear to be a fruitful investigation.

#### Gastrointestinal system

The effect of opioids on gut mobility among chronic opioids users are common and well known. A large study in France found that the prevalence of opioid-induced constipation was 21% with prolonged (>1 month) use [68]. Transdermal and partial agonists exhibit a lower incidence of this side effect. Use limited gut antagonist may partially reverse opioid-induced constipation [69]. The most characteristic feature of opioid-induced constipation is lack of development intolerance. Therefore, chronic opioid use has a significant negative impact on the quality of life but importance of these finding to perioperative care is somewhat limited.

Several mechanisms support metabolic disturbances in chronic opioid intake. Lipid profiles are often affected by the chronic use of opioids as exhibited by depression of HDL with concurrent elevation of LDL levels. While derangements in glucose metabolism often occur during acute use, no differences in glucose levels are seen in chronic users [36]. Since blood glucose control during the perioperative period is paramount, it remains to be seen if chronic drug abusers have higher rate of derangements in serum glucose potentially translating to less favorable outcomes for wound healing and surgical recovery.

#### Integumentary System

Opioids are a potent inducer of keratinocyte mobility and proliferation. In addition, μ receptor stimulation improves angiogenesis. These findings led to a clinical trial of topical morphine in order to augment wound healing. Both trials are underway. However, in small clinical study, patients who were exposed to narcotics exhibited less likelihood to heal chronic wounds [70]. The correlational nature of the study, coupled with the influence of the potential confounders, warrants further investigation in reconciling clinical findings with experimental observations.

### Clinical relevance

The most validated epidemiological data suggest an increased risk of cardiac death in a patient taking chronic opioids. Since the mechanism of this mortality excess is unclear, it is challenging to provide recommendations for perioperative specialists when faced with the increasingly common situation of a patient on chronic opioid therapy.

Immunosuppression is a significant and persistent side effect of prolonged narcotics used. However, the clinical translation of these findings is much less clear. The effect of opioids on cancer progression is long and fiercely debated. Immunosuppression triggered by exogenous opioids can be enabling for neoplasm emergence or re-occurrence. In addition, opioids stimulate angiogenesis by inducing expression of VEGF in the endothelium. The net effect should be promoting neo-plastic growth. This was demonstrated in xenotrans-plant animals with respect to breast cancer [71,72]. Retrospective analysis epidemiological data showed that patient treated with morphine suggests that in some cancer increased the dose of morphine is related to a less favorable outcome in breast cancer, rectal cancer, and other neoplastic disease [73–76]. However, the large prospective study failed to show any clinically significant effect in a breast cancer patient in a cohort study [77]. Considering that morphine is standard use to treat the patient long term, there is an urgency to check this effect inpatient population. Three strategies are available for the anesthesiologist to minimize the effect of morphine on tumor progression: use of the regional anesthesia, co-application of peripherally restricted opioids antagonists, and/or change in narcotics given. Any of these approaches need to be clinically verified.

The effect of prolonged opioids intake on seizure threshold, glucose metabolism or stress response has been demonstrated, but lack of clinical translation precludes providing a specific recommendation for anesthesia plan preparation in addition to ASA standards.

Finally, it is important to remain cognizant of the differential efficacy of opioid therapy according to sex. Although the mechanism of this difference has still yet to be elucidated, multiple clinical studies have shown that women frequently experience more pain during surgical procedures and often require more opioids to achieve similar analgesia to men [48,78–80]. Understanding the magnitude of this effect should guide perioperative specialists to manage patients accordingly in the postoperative period.

### Future Research Directions

Lack of well controlled, prospective studies conducted with patients is the most important recommendation for research progress in the future. Small studies with well-controlled confounders, with sound methodology (longitudinal series), can provide a high level of evidence leading to formulating a recommendation for future studies. Considering significant heterogeneity of opioids effect design in targeted studies may be more sensitive than traditional large cohort study involving several hundred subjects. Precision medicine may be an important factor in determining which patient may be disproportionally more affected by chronic uptake of opioids.

Clinical testing of the effect of different narcotics on the progression of the cancer is a potentially promising field for discovery. Considering that cancer patient is often on long term narcotics while fighting the neoplastic growth choice of the narcotics based on it immune-modulatory properties made be clinical sounds strategy. Proof of concept study is needed.

Metabolic effects of chronic opioids must be evaluated in conjunction with their hormonal properties. Finally, the effect of the blunted stress response on perioperative comorbidity should be explored since the laboratory studies consistently point to depressed ability to handle stress.

## Conclusion

Despite several years of research, we still possess a very limited understanding of all the potential effects of opioids on human health. Animal models and *in vitro* studies suggest potential effect but the complex nature of the neurohormonal-immunological interaction of chronic opioid intake warrants precision driven human studies. The promising direction of the anesthesiology-relevant discovery lies in understanding cancer progression, metabolic effects, and the ability to handle the acute stress in a patient taking opioids chronically.
